# Editorial and Miscellaneous

**Published:** 1861

**Authors:** 


### EDITORIAL.

*The Pathology and Treatment of Venereal Diseases. By reeman J. Bumstead, M. D.*, New York. Blanchard & Lea, Philadelphia.—On the announcement of this work, we were prepared for an addition to the catalogue of eminently useless works, but on its reception and perusal we return to the authors and publishers our most hearty thanks, for by far the most valuable contribution to this particular branch of practice that has seen the light within the last score of years.

The time worn notions and methods of practice contained in most of the so-called standard treatises upon this subject, every observant practitioner must have seen to be wholly inadequate and imperfect. We hesitate little in saying that no medical man, with a fair average of practice in this class of diseases, has found it possible to accept either the principles or treatment laid down in the vast majority of works on the subject—he certainly would have little cause to plume himself upon his success were he to attempt any such reliance.

The present work is brought up to the level of the time, by a clear sighted observer, of copious reading and large practical experience. Many of his views will strike those who have merely a book-knowledge of the subject as novel and perhaps as incredible, but experts will yield most of them immediate assent. Dr. Bumstead epitomizes in his preface the most important topics as follows:

"*The distinct nature of the two species of Chancre;* the innocuousness of the secretion of the infecting chancre, when applied to the person bearing it, or to any individual affected with the syphilitic diathesis; the removal of certain obstacles to a general belief in the contagiousness of secondary lesions; the fact that syphilis pursues the same course, whether derived from a primary or secondary symptom, commencing in either case with a chancre at the point where the virus entered the system; the definite period of incubation of the true chancre, and of general manifestations; the inefficiency of the abortive treatment of syphilis; and the phenomena of syphilization and their correct interpretation."

To say that the author has given a perspicuous and complete elucidation of all these remarkable ideas is not beyond what the book warrants. Yet those of our readers who have not kept pace with the investigations of the last decade upon this subject, will be a little startled at reading of the corroding venereal ulcer,—phagædenic perhaps—as totally distinct in nature from true syphilis. But the author to our mind proves, so far as can be done in such subjects, "the quality of syphilis." Myriad enigmas are unravelled by the process of developement of this idea. Hereafter we must recognize "the Chancroid and its Complications" as quite a different disease from the true hard, infecting chancre, "the initiatory lesion of acquired syphilis."

The first part of the work, which treats of "Gonorrhœa and its Complication," establishes the non-identity of Gonorrhœa and Syphilis; advocates the origination of true Gonorrhœa from the secretions of women not specifically diseased; the occurrence of gonorrhœal symptoms and even contagious discharge in the male without previous coitus; and we must confess upon these latter points indulges in latitudinarian views which are largely at variance with many preconceived opinions. It seems remarkable that so many escape infection.

Irrespective of the novelties in opinion contained in Dr. Bumstead's book, many of which may challenge discussion, his clear and accurate descriptions of the various forms of venereal disease, and especially the methods of treatment he proposes are worthy of the highest encomium. In these respects it is better adapted for the assistance of the every day practitioner than any other with which we are acquainted.—In variety of methods proposed, in minuteness of direction, guided by careful discrimination of varying forms and complications, we write down the book as unsurpassed. It is a work which should be in the possession of every practitioner.

---

*The Morbid Effects of the Retention in the Blood of the Elements of the Urinary Secretion. By W. W. Morland, M. D.* Fiske Fund Prize Essay. Blanchard & Lea, 1861, pp. 83.—This monograph, first presented to the Rhode Island Medical Society as a prize essay, then published in the American Medical Journal, was believed worthy of appearing in a more permanent shape, suitable for the library and reference, and the consequence is its appearance in thin octavo in the usual neat style of Blanchard & Lea. It is a valuable *resume* of observations upon this deeply interesting topic both original and collated. Whilst the greater proportion of the matter is to be found scattered throughout monographs and various systematic treatises, the author has done the profession essential service by bringing it together in compact form, and by careful study has separated much of the unreliable, or worse, which has been permitted to confuse the student. He discusses first the normal constituents of the secretion and then respectively the effects of each when unduly retained.—Nineteen pages are thus devoted to the retention of Urea;—about twenty pages to that of Uric Acid; six pages to Creatine, Creatinine and Hippuric acid; the same to Chlorine and Chloride of Sodium; four to Ammonia; and three to Iron and Uræmatine.

The treatise although brief, is quite complete, and no educatad physician can dispense with the information contained. Time has been when the stomach was considered the seat of about all diseases; the liver also has been charged with most of the disorders to which flesh is heir; but the kidneys, considering the well known importance of their functions, it is safe to say have not as yet attracted professional attention to a suitable extent. How many cases of convulsions, puerperal and other; how many cases of congestion of the brain and other organs, have owed their presence and fatal result to lesion or failure in action of the renal organs so little noticed, so little studied? We trust that general perusal of this essay may recall the attention of the profession to the fact that disease of these organs is not infrequent in occurrence and is always dangerous.

---

*Physician's Diary and Visiting List for* 1862. Lindsay & Blakiston. From W. B. Keen & Co., Lake Street. It is scarcely necessary to more than announce the issue of this now established medical "institution"—about every physician being familiar with its convenient character and arrangement.

Attention is directed by an Eastern cotemporary to a few omissions which it would be well for each purchaser to insert with his own pen. Among the list of antidotes *Tannin* should be recognized as antidotal to *Strychnia*, and the effects of *Liquor Potassæ* in neutralizing the narcotic effects of Belladonna should not be forgotten. In directions for restoration of suspended animation, it is also suggested that the Sylvester method has been again revived in preference to Marshall Hall's, which for a time had superseded it, and to this also might well be super-added the means recommended for the restoration of persons under the influence of Strychnina.

---

*Elmer's Hand-Book of Practice* for 1862, is received from the publishers, and it is filled as in former years, with matters of convenience and interest to every practitioner of medicine. Those who have once made use of it will hardly content themselves without it. It may be procured at S. C. Griggs & Co's.

---

*Map of Virginia.*—Mr. James T. Lloyd, the enterprising publisher, whose map of the Southern States we noticed in lithe last issue of the Journal, has laid us under renewed obgations by forwarding the official map of the eastern half of the State of Virginia to be soon followed, as he promises, by that of the Western half. It is the only full, complete and authoritative map published, and is beautifully executed.—We cordially recommend it to all interested, (and who is not?), in the stirring events now transpiring on the "sacred soil."

---

*Treatment of Fractures of the Long Bones, by simple Extension.* By John Swinburne, M. D., one of the Surgeons of the Albany City Hospital. From the author. This pamphlet of fifty pages is a reprint of a paper read by Dr Swinburne, before the New York State Medical Society at their last meeting, and which was briefly noticed in this Journal soon after. It is now put in a more accessible and permanent form, illustrated with appropriate diagrams, and we find bears re-reading excellently well. The author sustains his positions with great vigor and force. The importance of the subject demands the general attention of the profession. The simplicity of the apparatus recommended is not the least of its merits, although general adoption of the practice would sadly interfere with the profits of the splint makers, and rob the walls of the physician's office of the formidable array of paraphernalia with which after a few years of practice they are prone to be encumbered. Dr. Swinburne depends upon nature to supply splints and compresses in the shape of muscles, fasciæ, integument, etc. By his methods he claims that all varieties of fracture of the long bones can be as well treated in a country farm house, miles away from the splint makers, the carvers and moulders as in the best appointed hospital with its almost endless variety of instrumental devices.—In these days of prosecutions for *malapraxis*, every surgeon ought carefully to investigate every systematic method of treatment of fractures which promises exemption from deformities which will sometimes occur, notwithstanding employment of the approved methods heretofore in vogue.

*Report of Committee of the Boston Society for Medical Improvement on the alleged dangers which accompany the Inhalation of the vapor of Sulphuric Ether.*—Boston is still haunted with the ghost of the Letheon—the "hub" is still exercised in perpetual revolution about anæsthesia, its pet child, though it found its advent to the amphitheatric atmosphere in the questionable guise of a secret compound, under the joint and several proprietorship of Dentist Morton, of unhappy fame, and Prof. Jackson, of large chemical and Lake Superior experiences. The report culminates as follows :

The general conclusions which have been arrived at by your Committee may be summed up as follows:

1st. The ultimate offects of all anæsthetics show that they are depressing agents. This is indicated both by their symptoms and by the results of experiments. No anæsthetic should therefore be used carelessly, nor can it be administered without risk by an incompetent person.

2d. It is now widely conceded, both in this country and in Europe, that sulphuric ether is safer than any other anæsthetic, and this conviction is gradually gaining ground.

3d. Proper precautions being taken, sulphuric ether will produce entire insensibility in all cases, and no anæsthetic requires so few precautions in its use.

4th. There is no recorded case of death, known to the Committee, attributed to sulphuric ether, which cannot be explained on some other ground equally plausible, or in which, if it were possible to repeat the experiment, insensibility could not have been produced and death avoided. This cannot be said of chloroform.

5th. In view of all these facts, the use of ether in armies, to the extent which its bulk will permit, ought to be obligary, at least in a moral point of view.

6th. The advantages of chloroform are exclusively those of convenience. Its dangers are not averted by its admixture with sulphuric ether in any proportions. The combination of these two agents cannot be too strongly denounced as a treacherous and dangerous compound. Chloric ether, being a solution of chloroform in alcohol, merits the same condemnation.

It is clear that Boston cannot abide chloroform, although Dr. C. T. Jackson, one of the Committee, permits under quasi protest the use of a mixture of four parts ether and one part chloroform, as more convenient from lessened bulk for army purposes. We apprehend that notwithstanding the labored argumentation of the Committee that chloroform will still maintain its complete ascendency in general and army practice, as we most steadfastly believe it ought.—That the proposed mixture with ether secures no real advantage save a more gradual introduction of the chloroform into the system, a result which can more readily and pleasantly be secured by appropriate manner of exhibition. That in a large proportion of cases anæsthesia cannot be secured by ether without great expenditure of time, distress to patients, annoyance to attendants; excessive amounts of the vapor;—local irritation of the respiratory passages, rarely experienced from chloroform; a disordered state of the nervous system which retards recovery in a marked degree, and finally when anæsthesia is secured the danger from ether is no less than that from chloroform. An analysis of the real causes of reported death from chloroform according to the methods adopted by our Boston friends with reference to those reported from ether, would unquestionably largely diminish the proportion supposed attributable to the anæsthetic. The *post hoc ergo propter hoc* argument has been admitted to a most unreasonable extent. Before the introduction of any sort of anæsthetics surgical patients occasionally died in the most unaccountable manner, after comparatively slight operations.—All such cases are now incontinently charged upon the anæsthetic employed. But even with this disadvantage, anæsthetics have steadily urged their way into general use, and chloroform ranks the column. We opine that the present onslaught will not materially lessen the confidence of the profession in its right to the position.

———

*Quinine as a Prophylactic in Malarious Diseases.*—Medicine in several respects is quite like theology. It has been complained that all creeds and opinions can prove themselves orthodox, by judiciously selected texts from the bible; and so vast a number of medical reasoners can prove their positions right, to their own satisfaction at least, by a series of facts which everywhere are floating loosely in their neighborhood.

Just now the whole nation is agog about the war, and a great number of professional gentlemen with the rest are anxious to do something for the army. Prominently among the things proposed is that the grand army as it moves southward, shall be guarded from the malaria which does there abound, by daily ingestion by each individual of some five or more grains of quinine, and true to the method of reasoning suggested above, abundant instances are adduced where quinine has been in like manner taken as prophylactic with satisfactory results.

Let us look at the matter in an economical point of view at the outset. Of the army of five hundred regiments, it is a moderate estimate that one half will, if the war continues, be moved into the realms of Dixie and malaria, so that the prophylactic will be in daily requisition. Five grains to each person connected with a regiment, not including camp followers, teamsters, etc., will involve the use of, (in round numbers) ten ounces of quinine, or more exactly, about eleven of the ordinary ounce bottles, or for the two hundred and fifty regiments requiring its use, two thousand seven hundred and fifty bottles *per diem!* But discard seven hundred and fifty bottles for failures in exhibition, hospital patients, recusants, spiritualists, homœopathists, etc., and there still remains a nett 2000 bottles of quinine with all its well known imperfections on its head, to be purchased by the Government to be forced down the throats of our brave soldiery, *nolens volens.* And this to be continued eight or at least six months in the year— say three hundred and sixty thousand bottles. At the necessarily enhanced price of the article, the cost to the Government would fall little short of a Million of Dollars. Such an expenditure is not lightly to be made, to say the least of it.

It is scarcely more than a dozen or fifteen years since the doctrine was largely entertained in the malarious regions of the west that malaria penetrated and pervaded all things as a subtle poison, that it was held in solution in the water, suspended in the atmosphere, lurked in the finest roast of meat, and laid in ambush in the wholesomest looking loaf. All constitutions were impressed by it, and all diseases modified by it. To this wholesale assumption another was brought as a fellow, that Quina is the antidote to malaria wherever concealed and however manifesting its noxious influence.—The practical result was that all the adults took quina with their morning coffee, and the children with their bread and milk. History records its immense consumption and consequent enormous price. The writer has taken it daily for months in succession, with frequent recurring chills notwithstanding, until thirty grains became the ordinary and necessary anti-periodic dose. Scores and hundreds of western practitioners have seen its eventual failure as a prophylactic, and the rapid tolerance established. Quina is still largely and generally used, but with discrimination and judgment, and not under the guidance of the malarial and antidotal dogma. The rule now is Quina for the emergency—the paroxysmal development—other tonics and regimen for prophylaxis. The best of all prophylactics is good health, to be maintained by appropriate hygienic measures. So long as good health is present, there is little danger of attack by any of the multiform varieties of so called malarious disease. But let the causes of debility operate, and during the hours of depressionthe ague and its congeners are potent for evil. Comfortable clothing, dry feet, regular and sufficient sleep under shelter, good digestible food regularly eaten, a pint of good coffee both before and after standing guard; judicious medical attention to the condition of the digestive organs, and to occasional diseases—these and similar obvious methods will prevent more outbreaks of malarious disorder, than any mere medical prophylactic. Does not the medical profession yet see that the larger portion of its duty is not exhausted in inventing new drugs or the revival of obsolete methods of exhibition of old medicines?

---

*Ethical.*—An intelligent and respectable medical gentleman, whose diploma was received from one of our best Colleges, and whose claims to due professional recognition are further endorsed by letters from such eminent gentlemen as Prof. Pope, of St. Louis, has been for some time in reputable practice in this city. Somewhat startled by the peculiar tactics of a well known apostle of medical elevation and reform among us, he addresses to this journal a communication exhibiting the facts in a case of *consultation* (*!*) with said apostle, upon the 25th of October last. He somewhat mildly requests to know whether the apostolic course "is in accordance with the rules of Medical Ethics in this city—that it is not in conformity with Christian feeling, I am well aware."

The gist of the matter is that the apostle offensively ignored the family attendant, and insisted upon taking the case into his own hands, it being an obstetrical one, probably requiring, *to save the apostolic time*, instrumental interference. To obviate the degradation of a wrangle in the presence of the patient and friends, our correspondent, moved by the natural instincts of a gentleman, retired, leaving the apostle "master of the situation." He now appeals to the profession through our columns.

We prefer not to publish the communication, but present the facts as another leaf from the illustrated chronicles of medical elevation and reform. So many of the profession have submitted tamely to similar treatment from the same apostle, that we have lost faith in any general action. Our advice is, here and elsewhere: Never indulge in wrangling for right professional recognition in the chamber or house of the sick—it is unworthy of you as a gentleman; but, when next you meet the offender, compel him to apologize as becomes a gentleman; or, if he will not—we have no hopes of you, unless your thumb and forefinger become acquainted with his nose. *Verb. Sap.*

*Hydrochlorate of Ammonia.*—There is a remarkable similarity in the general effects of this salt and the Chlorate of Potassa. The Germans, especially, have long placed an estimate upon it as an alterative, which has surprised those who have not had considerable experience in its use. Lotions of varying degrees of strength of solution may, in the large proportion of cases, be advantageously substituted for the more expensive stimulating liniments usually directed. It thus forms a cheap and efficient rubefacient and discutient, well adapted for prescription in general prctice. In many forms of cutaneous disease, characterized by temporary improvement under the application of local remedies of a stimulating nature, it is particularly beneficial. Thus it is especially useful, both exhibited internally and employed externally, in connection with the arsenicals in treatment of original squamous affections, and where, in other varieties of tegumentary eruption, the furfuraceous surface is presented. Acne is often well controlled by its local application. As a discutient of slow glandular enlargements, it may rank with or next to Iodine, and has the advantage that it does not discolor the part of application.

As an alterative, liquefacient, resolvent, or promovent of cell and tissue metamorphosis, it is worthy of the high rank which our German confreres assign it—ordinarily improving digestion and renovating the secretions in a marked degree. Western practitioners, who have had much experience with it, given in full doses, in combination with Quina, assert that the system is left in a much better condition than when the latter is given singly, and that the necessity for administration of mercurial and other cathartics is very materially lessened or altogether removed.

The same peculiarities fit it for valuable exhibition in many obscure neuroses, where defective irregular innervation is the prominent symptom. We beg leave to recall the attention of the profession to this old remedy, as well worthy more frequent trial than it now receives, sensible, as we are, that, under judicious use, it will by no means as frequently disappoint in result as many newer and more fashionable medicines.

---

*The Army Examining Board.*—We are gratified at ascertaining that two of the members of the Examining Board, whose names we have not at hand, have been appointed Brigade Surgeons. The remaining three, it is announced, are appointed to the volunteer service of this State, with the rank of Majors. It is understood that the Legislature, during its next session, will legalize this appointment by the Governor, it being supposed to be the most economical method of providing for the payment of the Board. We regret to hear that the gentlemen composing the Board were somewhat annoyed at some remarks of this journal, in a previous issue; but congratulate ourselves that the appointment received from their gubernatorial patron may console them for such merely cutaneous inflictions. We trust that the harness of military rank may render their tegumentary tissue slightly more pachydermatous.

We are gratified in being able to announce that, by a coincidence of a remarkable character, since the appearance of our little paragraph before referred to, the examinations conducted have been of a much more practical sort, as well as more generally thorough upon those details which really require familiarity. We willingly award the merited meed, and will not detract from its radiance by claiming the slighest credit in the premises. Selected, as this Board was, by a non-professional man, irrespective of any general or individual expression of the profession, it is really quite remarkable that political and personal considerations should have permitted so capable a company of gentlemen to occupy so responsible a position. One or two of the physicians composing it, with whom we have had the honor of acquaintance, we are willing to endorse as everything desired in the post, and we sincerely trust that their eminent good nature (impervious even to the little pinch of the squib aforesaid,) may not allow them to approve candidates they do not find to be worthy and well qualified. We are, indeed, assured they will not, for we believe them fully alive to the importance of their position, and though perhaps occasionally a little sensitive to emotional influences, nevertheless thoroughly patriotic at heart.

---

*Army Med. Appointments in Michigan.*—Gov. Blair has exercised the appointing power vested in him, by selection of gentlemen of the most marked ability. We scarcely know of an instance where Michigan has Surgeons in the field but what are of the very cream of the profession. We are led to these observations by the recent choice of Dr. J. Andrews, of Paw Paw, a gentleman of high distinction, varied knowledge and large experience, as Surgeon to Col. Kellogg's Cavalry regiment, recently sent into the field of action. Michigan soldiers will not fail of excellent professional treatment, whatever their fortunes before the enemy or unaccustomed climate. Honor to Gov. Blair.

---

*Notice to Subscribers.*—A very considerable number of our exchanges have been compelled by the stringency of the times to suspend publication. Others have run in debt, hoping for better times in future to enable them to meet their liabilities. This journal has done neither, nor will it suspend or run in debt whatever the times. But some little irregularity of issue has been necessitated by the small proportion of receipts to expenditure. Our subscribers must pardon us this, as it will be seen to be dependent more upon them than upon us. There are large arrearages upon our books, and a large list of subscribers, but these have not paid the printer, paper-maker or binder, these have been mainly paid from our own pockets. Early in the year, during the decline and fall of Illinois and Wisconsin currency, our receipts were nominally considerable, but our losses upon bills received were very large by their depreciation, nevertheless we credited the major part at par. We have a goodly list of paying subscribers, and for the benefit of these, having determined upon prompt issue of each monthly number, we have concluded, it must be confessed with reluctance, to erase from our mail list a great number of names whose accounts have remained unadjusted year after year. Justice to paying subscribers and to ourselves demands this, and all interested will please to take due notice. We shall then take such steps as seem feasible for the collection of each of these old bills. Meanwhile, we request prompt transmission of all arrearages, whether of recent or long standing.

---

*Books for Sale.*—Any one wishing to procure a full set of the "American Journal of Medicine and Collateral Sciences," Edited by I. Hays, M. D., Philadelphia, from Jan. 1st, 1847, to the close of the present volume, 1861, well bound and in perfect preservation; or the "London Lancet," American reprint, from Jan., 1847, to 1855; or the "Boston Medical Journal," '47 to '57; or the first twelve parts of "Braithwaite's Retrospect," all well bound and in good order, can procure the same, at low figures, by application to the Editors of this Journal, or addressing a line to box 4458 Chicago P. O.

